# Directions vs. averages: an *in-vivo* comparison for cardiac DTI

**DOI:** 10.1186/1532-429X-17-S1-P25

**Published:** 2015-02-03

**Authors:** Andrew D Scott, Pedro Ferreira, Sonia Nielles-Vallespin, Laura-Ann McGill, Dudley J Pennell, David Firmin

**Affiliations:** 1 Cardiovascular Biomedical Research Unit, The Royal Brompton Hospital, London, UK; 2 National Heart and Lung Insitute, Imperial College, London, UK; 3 Intramural Research - National Heart Lung and Blood Institute, National Institutes of Health, Bethesda, MD, USA

## Background

The ability to interrogate cardiac microstructure has led to much recent interest in *in-vivo* cardiac diffusion tensor imaging (cDTI). However, when compared to studies performed in neuro-imaging, very little work has been done to determine the optimal diffusion encoding schemes. Previous work has suggested that accuracy is improved by increasing the number of diffusion encoding directions(N_dirs_)^1,2^, but comparisons in the heart have been limited to fixed animal specimens^3^. Here we compare parameters derived from cDTI using data acquired *in vivo* with an increasing N_dirs_.

## Methods

10 healthy subjects were imaged on a Siemens Skyra using the STEAM-EPI cDTI sequence^4^ in a short-axis slice of the mid left-ventricle with the optimal protocol recently described^5^ (b-values: 150 and 750 smm^-2^, 2.8x2.8x8mm^3^ resolution). This was repeated with N_dirs_=6, 10, 12 and 20 (standard Siemens product directions) and 12 averages (N_avs_) were acquired in each direction. The diffusion tensor and parameter maps including mean diffusivity (MD), helical angle (HA) and fractional anisotropy (FA) were calculated as previously described^5^, using all averages and all directions together to provide a reference data set. The processing was then repeated for each set of diffusion encoding directions with varying numbers of averages chosen to match the total images used N_tot_ = N_avs_ x N_dirs_, as closely as possible to 24, 36 and 60 and also using N_avs_=12.

## Results

**Figure 1:**
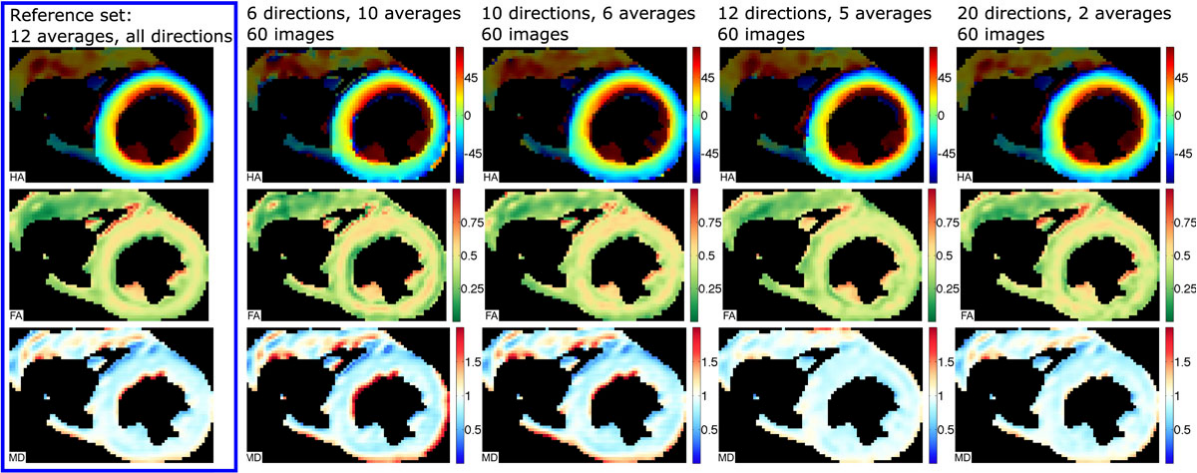
Maps of helical angle (top row), fractional anisotropy (middle) and mean diffusivity (bottom row) for one example healthy subject. Maps were calculated for one reference data set (left hand column) with all available data and then with combinations of N_dirs_ and N_av_ to total N_tot_=60 in each case. No consistent changes in parameter map were observed when altering N_dirs_.

**Figure 2:**
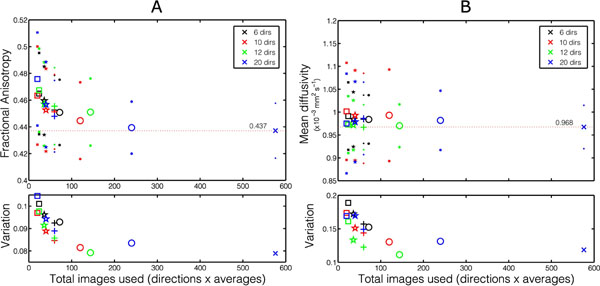
Mean values of fractional anisotropy (A) and mean diffusivity (B) for all 10 subjects (smaller points indicate standard deviations) in the left ventricle, colour coded by the N_dirs_ and plotted with the N_tot_ used in the tensor calculation. The horizontal lines indicate the mean values obtained from the reference data set which used every encoding direction and N_av_=12. The lower subplots show the standard deviation in the left ventricle averaged over all 10 subjects to provide some indication of the variability of the parameters within a subject. For a given N_tot_, the data acquired with N_dirs_=10 directions appears to most commonly have the least variation and the FA closest to the reference value. For MD, N_dirs_=12 appears to be optimal.

Figure 1 shows example parameter maps (HA, MD and FA) calculated using all directions (N_dir_=48) and all averages (N_av_=12) together compared to each diffusion encoding scheme processed with N_tot_=60. There was no consistently visible difference between the encoding schemes. Figure 2 shows mean MD and FA values for each N_dirs_ plotted with the N_tot_ and the average variation (standard deviation) in these parameter maps over the left ventricle. For a given N_tot_, N_dirs_=10 appears to have the minimum variation of FA across the left ventricle and most commonly has FA closest the reference value and N_dirs_=12 appears to be superior when considering MD. However, a comparison of MD and FA values when N_tot_=60, showed no statistically significant difference between N_dirs_ (1-way repeated measures ANOVA; MD: p=0.59; FA: p=0.82).

## Conclusions

While simulations in previous work have found increasing N_dirs_ to result in more accurate results, our results suggest that any resultant changes in MD or FA measured in *in-vivo* myocardium are small.

## Funding

This work was performed at The National Institute for Health Research Funded Cardiovascular Biomedical Research Unit at The Royal Brompton Hospital and Imperial College London.
